# Genomic analysis of *Agrobacterium radiobacter* DSM 30147^T^ and emended description of *A. radiobacter* (Beijerinck and van Delden 1902) Conn 1942 (Approved Lists 1980) emend. Sawada *et al.* 1993

**DOI:** 10.4056/sigs.4688352

**Published:** 2014-01-02

**Authors:** Linshuang Zhang, Xiangyang Li, Feng Zhang, Gejiao Wang

**Affiliations:** State Key Laboratory of Agricultural Microbiology, College of Life Sciences and Technology, Huazhong Agricultural University, Wuhan, 430070, P. R. China

**Keywords:** *Agrobacterium radiobacter*, non-phytopathogenic *Agrobacterium*, genome sequence, comparative genomics, emended species description

## Abstract

*Agrobacterium radiobacter* is the only known non-phytopathogenic species in *Agrobacterium* genus. In this study, the whole-genome sequence of *A. radiobacter* type strain DSM 30147^T^ was described and compared to the other available *Agrobacterium* genomes. This bacterium has a genome size of 7,122,065 bp distributed in 612 contigs, including 6,834 protein-coding genes and 41 RNA genes. It harbors a circular chromosome and a linear chromosome but not a tumor-inducing (Ti) plasmid. To the best of our knowledge, this is the first report of a genome from the *A. radiobacter* species. In addition, an emended description of *A. radiobacter* is described. This study reveals information that enhances the current understanding of its non-phytopathogenicity and its phylogenetic position within *Agrobacterium* genus.

## Introduction

*Agrobacterium radiobacter* DSM 30147^T^ (= ATCC 19358^T^) was first isolated from saprobic soil in 1902 as *Bacillus radiobacter* [1] and obtained its current name until *Agrobacterium* genus established by Conn in 1942 [2]. Based on phytopathogenic properties, Conn divided *Agrobacterium* into 3 species, *A. radiobacter*, *A. tumefaciens* and *A. rhizogenes* [2]. Subsequently, *A. rubi*, *A. vitis* and *A. larrymoorei* were also identified within the *Agrobacterium* genus [3-6]. Recently, *A. rhizogenes* was transferred to *Rhizobium* genus, as *Rhizobium rhizogenes*, based on multilocus sequence analysis (MLSA) using several housekeeping genes (*rrs*, *atpD* and *recA*) [7,8]. In addition, Young *et al.* proposed that *A. radiobacter* should have priority over *A. tumefaciens*, and *A. tumefaciens* may not officially represent a species [8,9]. Thus, currently, the genus *Agrobacterium* contains four validly named species, *A. radiobacter*, *A. vitis*, *A. rubi* and *A. larrymoorei* [7-9].

A taxonomic classification that relies on the phytopathogenic phenotypes may not accurately reflect the actual phylogenetic relationships of strains within *Agrobacterium* [10]. Accordingly, an alternative classification method was applied which divided most *Agrobacterium* strains into 3 biovariants (Biovars I, II and III) [10]. Among the 3 biovariants, Biovar I is the most complex group and includes several members (genomovars), designated as genomovar G1 through G9 and G13 [8,11]. At present, two strains in Biovar I have been completely sequenced: *Agrobacterium sp.* H13-3 (G1) and *A. tumefaciens* C58 (G8). The genome sequencing revealed that these strains contained two chromosomes and different numbers of plasmids. *A. radiobacter* DSM 30147^T^ also belongs to Biovar I (it is classified as a member of genomovar G4), which indicates its close relationship to *A. tumefaciens* C58 and *Agrobacterium sp.* H13-3 [12].

Most strains in the genus *Agrobacterium* are phytopathogens and induce crown gall tumors or hairy root diseases in their host plants [2]. However, *A. radiobacter* is an exception because it does not have the tumor-inducing (Ti) plasmid that contributes to the pathogenicity [13-16]. *A. radiobacter* members have been widely found in soil, in the rhizosphere of plants and in clinical specimens [17]. A strain of *A. radiobacter* was reported to enhance soil arsenic phytoremediation, indicating a potential application in bioremediation [18]. However, some members have been identified as opportunistic human pathogens [19]. So far, a total of 11 *Agrobacterium* genomes (3 finished and 8 draft genomes, listed in Table 1) have been sequenced but no genome of *A. radiobacter* has been reported. Considering its essential biological feature and important phylogenetic position in the genus *Agrobacterium*, we present the genome sequence of *A. radiobacter* DSM 30147^T^, the first sequenced strain in this species.

**Table 1:** General information and comparison of the 14 *Agrobacterium*-related genomes (12 *Agrobacterium* strains and 2 *Rhizobium* strains)

**Strain**	**Isolation source**	**Genome** **size (Mb)**	**CDSs** **#**	**Unique gene #**	**GenBank No.**
*A. radiobacter* DSM 30147^T^	Soil	7.18	6,834	548	ASXY00000000
*A. tumefaciens* str. Cherry 2E-2-2	Crown gall infected cherry rootstalk	5.43	5,040	482	APCC00000000
*A. tumefaciens* CCNWGS0286	Zinc-lead mine tailing	5.21	4,979	489	AGSM00000000
*A. albertimagni* AOL15	Hot Creek	5.09	4,811	734	ALJF00000000
*Agrobacterium sp.* 224MFTsu3.1	Plant-associated	4.80	4,593	141^a^	ARQL00000000
*R. lupini* HPC (L)	Saline desert soil	5.27	4,614	554	AMQQ00000000
*Agrobacterium sp.* ATCC 31749	Non plant-associated	5.46	5,529	984	AECL00000000
*A. tumefaciens* F2	Soil	5.47	5,288	2,070	AFSD00000000
*A. tumefaciens* 5A	Arsenic-enriched calciaquoll soil	5.74	5,517	539	AGVZ00000000
*Agrobacterium sp.* 10MFCol1.1	Rhizosphere	5.44	5,280	241^b^	ARLJ00000000
*Agrobacterium sp.* H13-3	Rhizosphere of *Lupinus luteus*	5.57	5,345	1,314	GCA_000192635
*A. vitis* S4	*Vitis vinifera*	6.32	5,389	870	GCA_000016285
*Rhizobium sp.* PDO1-076	Root of *Populus deltoids*	5.51	5,347	873	AHZC00000000
*A. tumefaciens* C58	Cherry tree tumor	5.67	5,355	196	GCA_000092025

^a, b^ Genomes were annotated through the RAST system [20]

The descriptions of *A. radiobacter* have been reported in 1902 [1], 1942 [2], 1980 [21] and 1993 [22]. After that, fatty acids and utilization of more carbon and nitrogen sources have been tested and showed that the major fatty acids (> 5%) are 16:0, 19:0 cyclo *ω*8*c*, summed feature 2 (one or more of 12:0 aldehyde, iso-16:1 I and 14:0 3-OH) and summed feature 8 (18:1*ω*7*c* and/or 18:1*ω*6*c*) [23]. The strain can utilize adonitol, D-fructose, D-galactose, D-mannitol, lactose and raffinose as sole carbon sources and L-ornithine, L-proline and L-serine as sole nitrogen sources [23]. Citrate utilization, nitrate reduction and urease are all positive [23]. In this study, we performed more physiological/biochemical analysis and present the emended description of *A. radiobacter*.

## Classification and features

**Figure 1:**
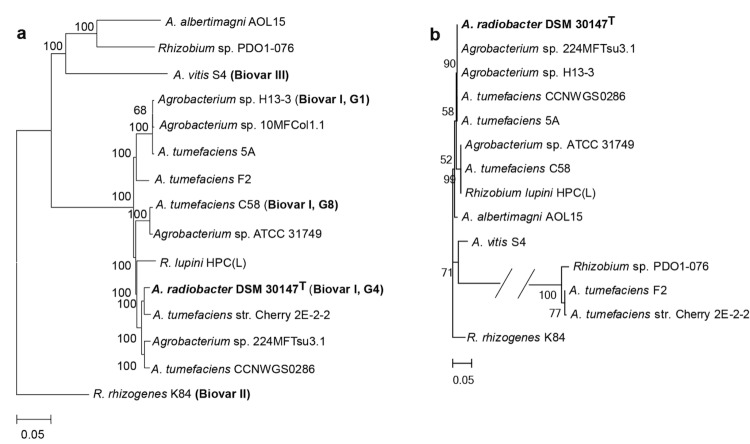
Phylogenetic trees highlighting the relationships among *A. radiobacter* DSM 30147^T^ and other closely related sequenced strains. (a) A tree was built based on 370 conserved proteins shared among the 15 genomes (12 *Agrobacterium* strains, 2 *Rhizobium* strains very closely related to *Agrobacterium* and one out-group strain, *R. rhizogenes* K84); (b) A tree inferred from the 16S rRNA genes of the same strains. The phylogenies were inferred by MEGA 5.05 using the neighbor-joining algorithm [20,24], and 1,000 bootstrap repetitions were computed to estimate the reliability of the branching order. The genome accession numbers of the strains used in the phylogenetic reconstructions: *A. albertimagni* AOL15, ALJF00000000; *Rhizobium sp.* PDO1-076, AHZC00000000; *A. vitis* S4, *A. radiobacter*, ASXY01000000; GCA_000016285; *Agrobacterium sp.* H13-3, GCA_000192635; *Agrobacterium sp.* 10MFCol1.1, ARLJ00000000; *A. tumefaciens* 5A, AGVZ00000000; *A. tumefaciens* F2, AFSD00000000; *A. tumefaciens* C58, GCA_000092025; *Agrobacterium sp.* ATCC 31749, AECL00000000; *R. lupini* HPC(L), AMQQ00000000; *A. tumefaciens* str. Cherry 2E-2-2, APCC00000000; *Agrobacterium sp.* 224MFTsu3.1, ARQL00000000; *A. tumefaciens* CCNWGS0286, AGSM00000000 and *R. rhizogenes* K84 GCA_000016265.

Genome sequences and 16S rRNA genes were used for phylogenetic analysis. In view of the close evolutionary relationship and the inconsistent phylogeny between *Agrobacterium* and *Rhizobium* [12], we pre-analyzed all sequenced strains in these two genera and found that two “*Rhizobium”* members were very closely related to the 12 *Agrobacterium* members (including strain DSM 30147^T^). Thus, all of the 12 *Agrobacterium* members with sequenced genomes, two *Rhizobium* strains [*R. lupini* HPC(L) and *Rhizobium sp.* PDO1-076] (Table 1) and an out-group strain *R. rhizogenes* K84 [7,8], were included in the phylogenetic analysis. A comparison of the 15 genomes revealed a total of 370 proteins that were shared across these genomes. A rooted neighbor-jointing (NJ) phylogenetic tree was constructed based on the shared amino acid sequences. As shown in Figure 1a, *A. radiobacter* DSM 30147^T^ was in the same cluster as the Biovar I members *Agrobacterium sp.* H13-3 (G1) and *A. tumefaciens* C58 (G8), and showed the closest relationship with *A. tumefaciens* str. Cherry 2E-2-2. A NJ phylogenetic tree was also constructed based on the 16S rRNA genes (Figure 1b). When comparing the trees generated by the core protein sequences with those generated by 16S rRNA gene sequences, small topological differences in topology were found between them. In comparison to the tree generated using the 370 conserved proteins, some strains could not be distinguished with a high degree of clarity using the 16S rRNA genes. Therefore, phylogenomic analysis was considered a more robust approach than that using the 16S rRNA genes to infer the phylogeny, especially for closely related strains [21,25,26].

**Figure 2:**
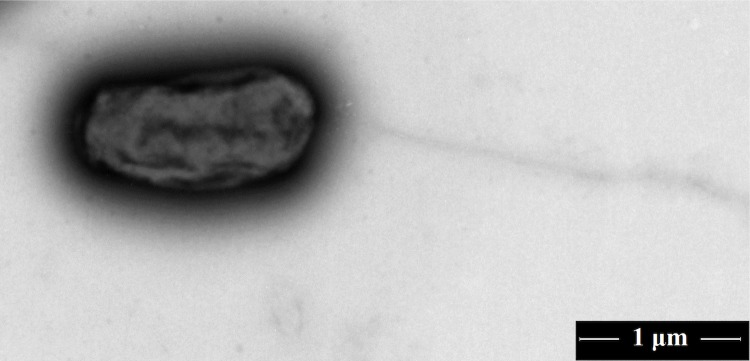
A transmission micrograph of *A. radiobacter* DSM 30147^T^, using 200 kV transmission electron microscopy FEI Tecnai G^2^ 20 TWIN (USA). The scale bar represents 1 μm.

Strain DSM 30147^T^ is rod-shaped (0.6-0.8 x 1.5-1.8 μm) (Figure 2). The enzyme activities and carbon sources utilization of strain DSM 30147^T^ were tested using API ZYM, API 20 NE and API ID 32 GN systems and the results are shown in Table 2 and in the emended description of *A. radiobacter.*

**Table 2:** Classification and general features of *Agrobacterium radiobacter* DSM 30147^T^ according to the MIGS recommendations [27,28]

**MIGS ID**	**Property**	**Term**	**Evidence code**
	Current classification	Domain *Bacteria* Phylum *Proteobacteria* Class *Alphaproteobacteria* Order *Rhizobiales* Family *Rhizobiaceae* Genus *Agrobacterium* Species *Agrobacterium radiobacter* type strain DSM 30147^T^	TAS [29] TAS [28] TAS [30,31] TAS [30,32] TAS [21,33] TAS [2,21,22,33-35] TAS [21,22,33] TAS [1-3]
	Gram stain	negative	TAS [22]
	Cell shape	rod-shaped	TAS [22]
	Motility	motile	IDA
	Sporulation	non-sporulating	TAS [22]
	Optimum temperature	25-28 ºC	TAS [22]
	Carbon source	arabinose, D-glucose, D-melibiose, D-ribose, D-sorbitol, gluconate, histidine, 4-hydroxybenzoate, 3-hydroxybutyrate, inositol, 2-ketogluconate, L-alanine, L-fucose, L-lactate, L-proline, L-rhamnose, malate, maltose, mannitol, mannose, N-acetyl glucosamine, propionate, salicin, sodium acetate and sucrose	IDA
	Energy source	chemoorganotroph	TAS [22]
	Terminal electron receptor	molecular oxygen	TAS [22]
MIGS-6.2	pH	6-7	TAS [22]
MIGS-22	Oxygen	aerobic	TAS [22]
MIGS-15	Biotic relationship	free-living	NAS
MIGS-14	Pathogenicity Biosafety level	non-phytopathogenic level 1, in individual cases, some members of this species are suspected human pathogens	TAS [36]
MIGS-4	Geographic location	not reported	
MIGS-5	Sample collection time	1902	TAS [1]
MIGS-4.1 MIGS-4.2	Latitude Longitude	not reported not reported	
MIGS-4.3	Depth	not reported	
MIGS-4.4	Altitude	not reported	

Evidence codes - IDA: Inferred from Direct Assay; TAS: Traceable Author Statement; NAS: Non-traceable Author Statement. These evidence codes are from the Gene Ontology project [37]. If the evidence is IDA, then the property was directly observed for a live isolate by one of the authors or an expert mentioned in the acknowledgements.

## Genome sequencing and annotation

### Genome project history

To make a comprehensive genomic comparison for the *Agrobacterium* genomes, the whole genome sequence of *A. radiobacter* DSM 30147^T^ was determined. This draft genome sequence has been deposited at DDBJ/EMBL/GenBank under accession number ASXY00000000. The version described in this study is the first version, ASXY01000000. The project information is summarized in Table 3.

**Table 3:** Project information

**MIGS ID**	**Property**	**Term**
MIGS-31	Finishing quality	High-quality draft
MIGS-28	Libraries used	Illumina Paired-End library (300 bp insert size)
MIGS-29	Sequencing platform	Illumina Hiseq2000
MIGS-31.2	Sequencing coverage	196.3 ×
MIGS-30	Assemblers	SOAPdenovo v1.05
MIGS-32	Gene calling method	GeneMarkS^+^
	GenBank date of release	July 12, 2013
	NCBI project ID	ASXY00000000
MIGS-13	Source material identifier	DSM 30147^T^
	Project relevance	Genome comparison

### Growth condition and DNA isolation

*A. radiobacter* DSM 30147^T^ was grown aerobically in LB medium [38] at 28 °C for 24 h. The DNA was extracted, concentrated and purified using the QiAamp kit according to the manufacturer’s instruction (Qiagen, Germany).

### Genome sequencing and assembly

Illumina Hiseq2000 with the Paired-End library strategy (300 bp insert size) was used to determine the whole-genome sequence of *A. radiobacter* DSM 30147^T^ and obtained a total of 15,140,909 reads (1.41 Gb data). The detailed methods of library construction and sequencing can be found at Illumina’s official website [39]. Using SOAPdenovo v1.05 [40], these reads were assembled into 612 contigs (> 200 bp) with a genome size of 7,122,065 bp and an average coverage of 196.3 ×.

### Genome annotation

The draft genome of *A. radiobacter* DSM 30147^T^ was annotated using the National Center for Biotechnology Information (NCBI) Prokaryotic Genome Annotation Pipeline (PGAP) [41], which combines the gene caller GeneMarkS^+^ [42] with the similarity-based gene detection approach. Protein function classification was performed by searching all the predicted coding sequences of strain DSM 30147^T^ against the Clusters of Orthologous Groups (COGs) protein database [43] using Blastp algorithm with *E-value* cutoff 1-e^10^.

## Genome properties

**Figure 3:**
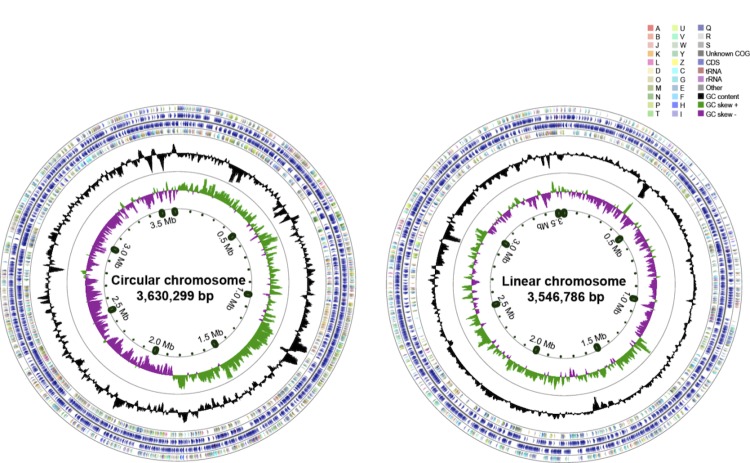
The circular representation of the *A. radiobacter* DSM 30147^T^ circular chromosome (left) and linear chromosome (right). From outside to center, ring 1, 4 show protein-coding genes colored by COG categories on forward/reverse strand; ring 2, 3 denote genes on forward/reverse strand; ring 5 shows G+C% content plot, and the innermost ring shows GC skew.

The whole genome of *A. radiobacter* DSM 30147^T^ is 7,122,065 bp in length, with an average GC content of 59.9%, and distributed in 612 contigs. Compared to the complete reference genome *A. tumefaciens* C58 [44] (also belonging to Biovar I,Figure 1), the whole genome of strain DSM 30147^T^ could clearly be divided into 2 replicons, a circular chromosome and a linear chromosome (Figure 3). In accordance with its non-phytopathogenicity phenotype, strain DSM 30147^T^ did not contain a Ti plasmid. Of the 6,894 genes predicted, 6,853 were protein-coding genes (CDSs), and 41 RNA genes. A total of 5,320 CDSs (77.85%) were assigned with putative functions, and the remaining proteins were annotated as the hypothetical proteins. The genome properties and statistics are summarized in Table 4 and Figure 3. The distribution of the genes into COG functional categories is shown in Table 5.

**Table 4:** Genome statistics

**Attribute**	**Value**	**% of Total**
Genome size (bp)	7,177,085	100
Number of contigs	612	
Contig N50	24,130	
Number of replicons	2	
Extrachromosomal elements	Unknown	
Total genes	7,151	100
Protein-coding genes	6,834	95.57
Pseudo genes	276	3.86
RNA genes	41	0.57
rRNAs	4	
Frameshifted genes	95	
DNA coding region (bp)	6,197,065	86.34
Protein-coding genes with function prediction	5,320	77.85
Protein-coding genes assigned to COGs	5,333	78.04
Protein-coding genes with conserved domain	5,986	87.59
Protein-coding genes with transmembrane helices	1,899	27.79
Protein-coding genes with signal peptides	550	8.05

**Table 5:** Number of protein-coding genes associated with the general COG functional categories in *A. radiobacter* DSM 30147^T^ genome

**Code**	**Value**	**% age**	**Description**
J	184	2.69	Translation, ribosomal structure and biogenesis
A	0	0.00	RNA processing and modification
K	461	6.75	Transcription
L	157	2.30	Replication, recombination and repair
B	0	0.00	Chromatin structure and dynamics
D	39	0.57	Cell cycle control, cell division, chromosome partitioning
Y	0	0.00	Nuclear structure
V	75	1.10	Defense mechanisms
T	284	4.16	Signal transduction mechanisms
M	282	4.13	Cell wall/membrane/envelope biogenesis
N	99	1.45	Cell motility
Z	0	0.00	Cytoskeleton
W	0	0.00	Extracellular structures
U	100	1.46	Intracellular trafficking, secretion, and vesicular transport
O	197	2.88	Posttranslational modification, protein turnover, chaperones
C	336	4.92	Energy production and conversion
G	585	8.56	Carbohydrate transport and metabolism
E	757	11.08	Amino acid transport and metabolism
F	115	1.68	Nucleotide transport and metabolism
H	224	3.28	Coenzyme transport and metabolism
I	188	2.75	Lipid transport and metabolism
P	481	7.04	Inorganic ion transport and metabolism
Q	148	2.17	Secondary metabolites biosynthesis, transport and catabolism
R	684	10.01	General function prediction only
S	546	7.99	Function unknown
-	1501	21.96	Not in COGs

## Comparative genome analysis of *A. radiobacter* DSM 30147^T^ with the other related genomes

Strain DSM 30147^T^ has the largest genome size of the 12 *Agrobacterium* strains sequenced to date and is larger than the 2 very closely related *Rhizobium* strain genomes as well (Table 1). OrthoMCL [45] was used to perform orthologs clustering analysis for the 14 genomes (Table 1). The results indicate that *A. radiobacter* DSM 30147^T^ shares 1,636 genes with the other 13 strains and contains 548 strain-specific genes (Table 1), which potentially encode products that contribute to species-specific features differentiating *A. radiobacter* from other *Agrobacterium* species [46]. In addition, on average, only 31% core genes were shared among the 14 genomes, which reveals a high-degree of diversity within *Agrobacterium* genus.

## Emended description of *Agrobacterium radiobacter* (Beijerinck and van Delden 1902) Conn 1942 (Approved Lists 1980) emend. Sawada *et al.* 1993

This emended description is based on that given by Beijerinck and van Delden 1902, Conn 1942 (Approved Lists 1980) and Sawada et al. 1993 with the following changes. Positive results are observed for acid phosphatase, α-glucosidase, alkaline phosphatase, arginine dihydrolase, β-glucosidase, citrate utilization, esterase (C4), leucine arylamidase, N-acetyl-β-glucosaminidase, naphthol-AS-BI-phosphohydrolase, nitrate reduction, urease and valine arylamidase, but negative results for α-galactosidase, α-mannosidase, β-fucosidase, β-galactosidase, β-glucuronidase, chymotrypsin, cystine arylamidase, esterase lipase (C8), lipase (C14) and trypsin. Arabinose, D-glucose, D-melibiose, D-ribose, D-sorbitol, gluconates, histidine, 4-hydroxybenzoate, 3-hydroxybutyrate, inositol, 2-ketogluconate, L-alanine, L-fucose, L-lactate, L-rhamnose, malate, maltose, mannose, N-acetyl glucosamine, propionate, salicin, sodium acetate and sucrose source while cannot assimilate adipate, caprate, 3-hydroxy-benzoate, itaconic acid, glycogen, 5-ketogluconate, phenylacetate, potassium, sodium malonate, suberate and valerate are utilized as the sole carbon sources. L-ornithine, L-proline and L-serine are utilized as nitrogen sources. The major fatty acids (> 5%) are 16:0, 19:0 cyclo *ω*8*c*, summed feature 2 (one or more of 12:0 aldehyde, iso-16:1 I and 14:0 3-OH) and summed feature 8 (18:1*ω*7*c* and/or 18:1*ω*6*c*). The members of this species are nonphytopathogenic, but in individual cases, some members of this species are detected as possible human pathogens.
